# Editorial: Recent advances in mitochondrial dysfunction and therapeutics for neurodegeneration and aging

**DOI:** 10.3389/fncel.2025.1650938

**Published:** 2025-07-15

**Authors:** Monokesh K. Sen, Keagan Dunville, Nicole Miles, Michelle Newbery, Neville S. Ng, Lezanne Ooi

**Affiliations:** ^1^Faculty of Medicine and Health, Charles Perkins Centre, School of Medical Sciences, The University of Sydney, Sydney, NSW, Australia; ^2^Kavli Institute for Systems Neuroscience and Centre for Algorithms of the Cortex, Norwegian University of Science and Technology (NTNU), Trondheim, Norway; ^3^Molecular Horizons and School of Chemistry and Molecular Bioscience, University of Wollongong, Wollongong, NSW, Australia

**Keywords:** mitochondria, neurodegeneration, Alzheimer's disease, Parkinson's disease, motor neuron disease

## Introduction

**Figure 1:**
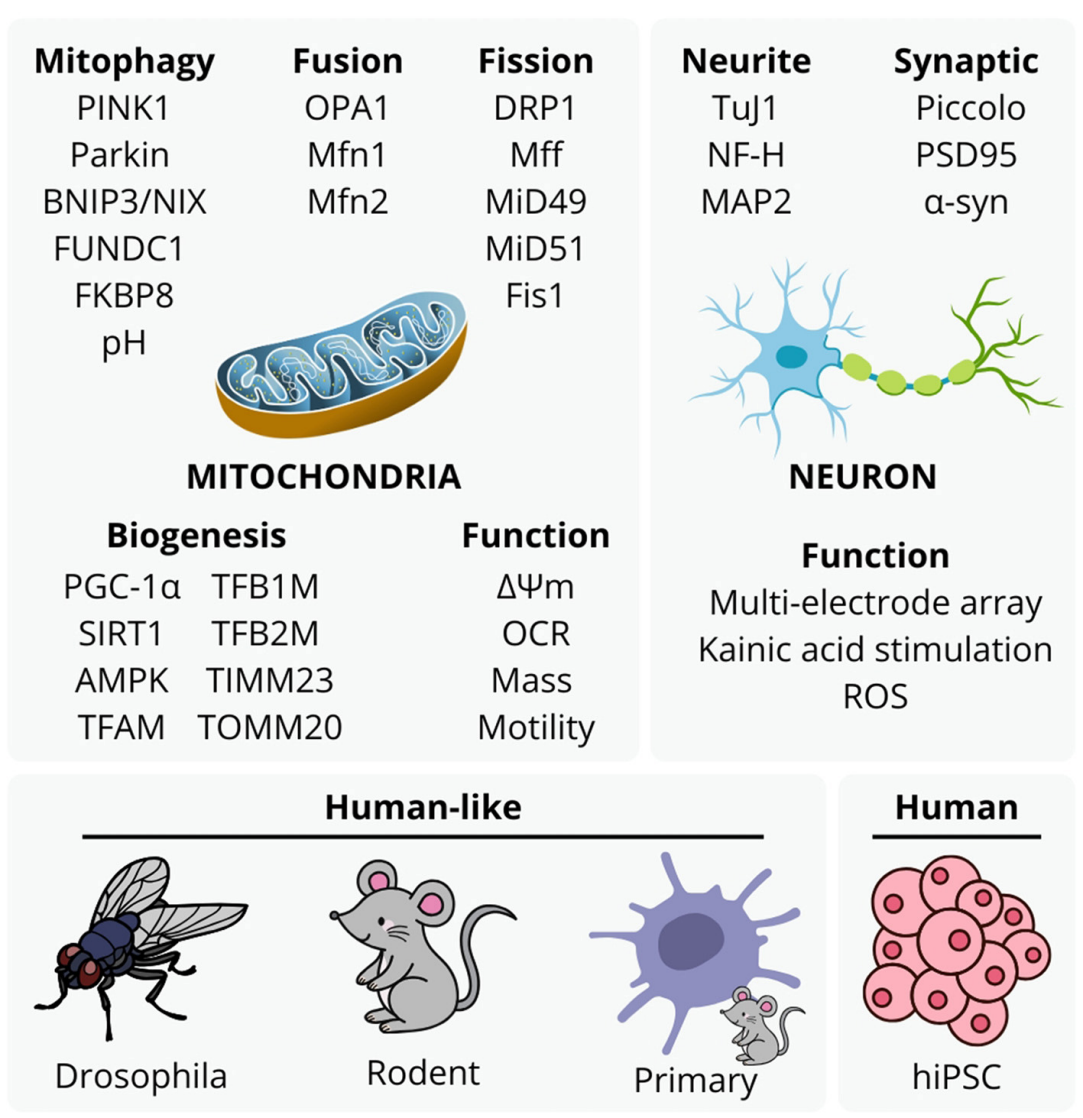
Experimental approaches to mitochondrial etiology with mitophagy, mitochondrial fusion, fission, biogenesis, function, and neuronal structure and function, utilized by studies in this topic. These are representative of typical approaches to analyse mitochondrial function in neurodegenerative disease and aging studies, although the accessibility and value of human stem cell -derived models with aspects animal ethics and validity continues to emerge with methodology of more complex models.

Recent estimates indicate that neurological conditions affect over 3.4 billion individuals globally (Steinmetz et al., 2024). A well-established contributor to neurological conditions is the decline of mitochondrial function, responsible for producing over 90% of cellular ATP via oxidative phosphorylation. Mitochondrial dysfunction is closely linked to cellular pathology, and cognitive and motor decline symptoms in neurodegeneration and aging. Common features of mitochondrial dysfunction include excessive reactive oxygen species (ROS) generation, damage to mitochondrial DNA (mtDNA), impaired mitochondrial quality control, and disrupted mitochondrial dynamics (Ng et al., 2024). Subsequent cellular damage includes disrupted protein synthesis, transport, and degradation, increased inflammation, oxidative damage to genomic and mitochondrial DNA, lipids, and proteins, aberrant calcium signaling, telomere shortening, senescence, eventual cell death (Ng et al., 2024), contributing to complex protein-nucleic acid mass aggregation and systemic inflammation. Submissions to this *Special Topic* collection exemplify a range of experimental approaches to analyse mitochondrial etiology in neurological disorders and aging (Figure 1). These studies highlight the significance of mitochondrial damage, dysfunctional mitochondrial biogenesis and mito-lysosomal clearance, and subsequent neuroinflammation in neurodegenerative diseases and aging, discussed in concluding notes of this editorial.

## Using iPSC neuronal models with microfluidics to analyze LRRK2 mutation in Parkinson's disease

Valderhaug et al. explored a pilot of a multichambered microfluidic slide and microelectrode arrays, using a Parkinson's disease induced pluripotent stem cell (iPSC) derived line with a *LRRK2* G2019S mutation, associated with both familial and sporadic forms, compared against its isogenic control. This combination allowed for the structuring of intrinsic network firing behavior, mitochondrial membrane potential, kymography, and oxidative stress, with and without the ionotropic glutamate agonist kainic acid. Neurons with the *LRRK2* G2019S mutation showed greater neurite outgrowth, increased mitochondrial motility that was decreased with kainic acid stress, decreased mitochondrial anterograde movement, and hyperpolarisation in LRRK2 G2019S networks. While observations are limited a single isogenic control, these data demonstrate a multimodal approach to conveniently analyse neurite density, electrophysiology and mitochondria.

## Effect of *SNCA* overexpression and *PRKN* in Drosophila melanogaster

A hallmark of Parkinson's disease is the aggregation of incorrectly folded α-synuclein (encoded by the *SNCA* gene) protein, as well as loss of function of parkin (encoded by *PRKN*), an E3 ubiquitin ligase. Mutations in *SNCA* and *PRKN* cause impaired mitochondrial morphology, resulting in dopaminergic neuron loss. Narwal et al. used a humanized Drosophila model to examine the effect of overexpressing *SNCA* and downregulating *PRKN* in dopaminergic neurons of the posterior brain with a GAL4/UAS system. Overexpression of *SNCA* and *PRKN* downregulation reduced dopaminergic neuronal clusters leading to progressive locomotor dysfunction. Altered mitochondrial morphology and fragmentation assessment utilizing the UAS-MitoGFP fly strain, occurred in a cluster-specific manner, affecting only a subset of dopaminergic neurons. *SNCA* overexpression also downregulated *PRKN*, not dissimilar to expected observations in Parkinson's disease. The study provides insights into the molecular interactions between α-synuclein and parkin and their impact on dopaminergic neurons. In particular, these data demonstrate eukaryotic conservation of the potentially toxic role of excessive α-synuclein expression, or downregulation of *parkin* and mitochondrial biogenesis.

## Mitochondrial role in sensory and associative cortical systems

Mitochondria play a pivotal role in neurodevelopment, and in this special topic collection, several groups investigate the role of mitochondria in sensory and associative cortical systems. Cavestro et al. investigate the role of Coenzyme A synthase (protein: CoA synthase, encoding gene: *Coasy*, Chr:11) in mouse *in vitro* and *in vivo* knockout models to dissect nuclear-mitochondrial crosstalk relevant to CoA-related disorders. CoA synthase loss-of-function mutations drive rare yet debilitating neurodegeneration, such as Coasy protein-associated neurodegeneration, and neurodevelopmental disorders, including Pontocerebellar Hypoplasia Type 12. Conditional knockouts were generated using a humanized glial fibrillary acidic protein (GFAP) promoter and *Coasy*^flox/flox^ mice, ensuring loss of CoA synthase in astroglial lineage cells. Intriguingly, GFAP-Coasy mice manifested two distinct, sex-independent phenotypes with motor deficits: early onset (euthanized by postnatal day 20) and late onset (unaffected mortality). Additionally, GFAP-Coasy mice exhibited severe layering deficits in the cortex and cerebellum, demonstrating that embryonic expression of CoA synthase is necessary for inherent cytoarchitectural establishment. Moreover, astroglial-specific expression of nuclear receptor coactivator 4 was decreased, coincident with increased lipid peroxidation and decreased mitochondrial respiration in GFAP-Coasy-derived primary astrocytes. Given that astroglial populations do not fully mature until postnatal weeks 3-4 in mice, deficits in astroglial cells are likely latent and do not fully account for lamination deficits. However, GFAP is also expressed by neural stem cells, thus deletion of CoA synthase may have occurred across all neural cell types, similar to the Nestin-Cre mouse model (Lagace et al., 2007). Regardless, this study suggests subpopulation variability with respect to CoA synthase expression and opens novel insights regarding nucleus-mitochondria crosstalk relevant to age-variant onset *Coasy*-linked disorders.

In the sensory system, Miwa et al. investigated a global axis between increased substrate consumption of N1-methylnicotinamide (MNAM), a byproduct of NAD^+^ biosynthesis and inhibitor of nicotinamide N-methyltransferase (NNMT), Sirtuin 1 (protein: SIRT1, encoding gene: *Sirt1*, chr:10), an NAD-dependent transcription factor-deacetylase and effector of NNMT, and their relevance to age-related hearing loss in mouse cochlea. Mice were fed a low-fat diet (LFD) or a low-fat diet with 1% MNAM supplemented from postnatal week 4 and were analyzed for whole body biometrics, auditory brain stem responses, and cochlear metabolomic analysis at 3 months, 6 months, and 12 months of age. The authors report that LFD alone induced steady weight gain over 12 months, whereas LFD + MNAM-fed mice lost significant weight, observable by month 6, although there were no significant changes in blood biomarkers. LFD + MNAM mice also exhibited significant hearing loss by month 6, evident by the increased decibel threshold required to evoke auditory brainstem responses and outer hair cell dysfunction. Histological quantification revealed decreased hair cell number, decreased spiral ganglion cell number, and decreased cochlear turning. Sirt1 protein level was also increased in LFD + MNAM cochlea, and together with the metabolomics analysis of 6-month-old mice, demonstrates notable dysfunction in the metabolic profile by a severe increase in pyruvate compared to the LFD mice. Considered together with previous Sirt1-knockout hearing loss models, this study provides evidence that cochlear physiological function is highly correlated with nuclear-mitochondrial communication, primarily mitigated through Sirt1 signaling axes.

## The role of diet in modulating mitochondria

Morgan et al. investigated the effects of a ketogenic diet, typically comprising 70% fat, 20% protein, and 10% carbohydrates, on mitophagy, a selective form of autophagy responsible for eliminating damaged or excess mitochondria. Using mito-qc mice, which express a pH-sensitive mCherry-GFP tag on the outer mitochondrial membrane, the researchers assessed mitophagy by quantifying mitolysosomes in retinal ganglion cells (RGCs) through immunoblotting with RNA Binding Protein with Multiple Splicing (RBPMS). The results revealed a significant increase in mitolysosome numbers in RGCs following ketogenic diet administration, while Müller glia - the principal glial cells in the retina - remained unaffected. Mechanistically, hypoxia triggers mitophagy and that the ketogenic diet serves as an additive enhancer of this process in retinal ganglion cells. The differing response of RGCs and Müller glial cells on ketogenic diet suggesting the variations in their metabolic requirements.

## Mitochondrial role in brain injury

In a literature review, Olatona et al. highlighted the critical role of mitochondria in neurodegeneration of post-traumatic brain injury (TBI), in particular the role of calcium homeostasis, metabolic dysregulation, oxidative stress, and neuroinflammation. The authors postulate that excessive glutamate release during injury results in hyperexcitability, in part due to failure of mechanically stimulated astrocytes to retain and metabolize glutamate. Excess calcium influx results in mitochondrial permeability transition pore (mPTP) formation with elevation of cytosolic calcium, disruption of electron transport chain, and reactive oxygen species production, followed by systemic release upon rupture, including molecular damage associated patterns contributing to neuroinflammation by astrocytes and microglia (e.g. ATP to purinergic receptors, cytochrome C to TLR4 receptors and mtDNA to TLR9 receptors). The interference of mitochondrial respiration persists through the initial response of hyper-excitability and hyper-glycolysis in the first several hours to days followed by hypometabolic phase for weeks, explored in human patients and rodent head impact models. Meanwhile the tissue damage is not restricted to areas of altered bioenergetics. They note similar mechanisms occur in progressive neurodegeneration such as Alzheimer's disease, Parkinson's disease, and motor neuron disease. The authors postulate that targeted therapies to mitochondrial biogenesis such as PGC-1α, or purinergic receptor and STING pathway inhibition may mitigate mitochondrial dysfunction in acute and chronic neurodegeneration.

## Mitochondrial biogenesis, fragmentation and quality control

Araujo et al. investigated the role of astrocytic mitochondrial fragmentation in a cell culture and murine model in age-related decline. They observed that primary senescent astrocytes displayed an increased number of mitochondria, decreased mitochondrial size (MitoTracker) and mitochondrial membrane potential (JC-1). Senescent astrocytes showed increased mitofusin 1 and 2, optic atrophy OPA2, DRP1 and Fis1 gene and protein expression. Young (3-4 months) and aged (over 18 months) mice were used to assess mitochondrial changes in aged astrocytes. In cells isolated from young and old mice, mitochondria were stained using TOMM20 in both astrocytes (GFAP+) and neurons (β-Tubulin III) by flow cytometry. Astrocytes and neurons from aged mice showed a decrease in TOMM20 fluorescence intensity. Aged animals show a decline in mitochondrial content in both astrocytes and neurons. Genes that regulated mitochondrial biogenesis, showed a reduction in Peroxisome proliferator-activated receptor gamma coactivator 1-alpha (PGC1α), Transcription Factor B1, Mitochondrial (TFB1), ATP Synthase F1 Subunit Alpha (ATP5A1) expression. Aged animals exhibit a decline in mitochondrial content in both astrocytes and neurons, as well as decreased expression of genes that regulate mitochondrial biogenesis, and increased DRP1 expression and mitochondrial fission in astrocytes. Astrocytes in aged mice showed a reduction in mitochondrial area, perimeter and a decrease in the number of elongated mitochondria. These data indicate that aging promotes mitochondrial fragmentation in the mammalian brain, impairs energy regulation and highlights a potential imbalance in fission and fusion within senescent cells.

Liu et al. extensively reviewed mitochondrial dysfunction with a focus on mitochondrial quality control processes, in particular biogenesis and mitophagy pathways. The authors highlight further studies could focus on PINK1/Parkin mitophagy validation *in vivo*, with challenges in specificity and blood-brain barrier permeability currently limiting clinical translation. Controversies persist regarding the significance and roles of mitophagy receptors, and their impact on mitochondrial dynamics (i.e. whether Drp1 consistently regulates and is required for PINK1/Parkin mitophagy, whether PINK1/Parkin mitophagy occurs *in vivo*, and role of isoforms of OPA1). Nonetheless we note the significance of neurodegeneration as proteinopathies remains, in some instances preceded by defective mitochondrial clearance. This is reinforced as the authors summarize that upregulated mitophagy levels can be protective in reducing accumulation of Aβ and hyperphosphorylated tau in Alzheimer's disease, α-syn in Parkinson's disease, mutant Htt in Huntington's disease and SOD1 or TPD-43 early in motor neuron disease progression, although potentially detrimental in latter stages of motor neuron disease. Direct or indirect impaired mitochondrial biogenesis as evident from loss of expression or activity in patient tissue, is thought to contribute to progressive cell death. The authors advise mitochondrial quality control can be further studied with multi-omics, single-cell analysis, and advanced gene technologies, crosstalk between mitochondria and other organelles (e.g., lysosomes, autophagosomes), which contribute to a vicious cycle of dysfunction in neurodegeneration.

## Conclusion

The studies compiled in this Research Topic observed various aspects of mitochondrial function in Drosophila, rodent, primary neural and human stem cell platforms, analyzing metabolism, mitochondrial biogenesis and mitophagy, neuroinflammation, electrophysiology. On the other, we note that mitochondrial dysfunction may inevitably coincide with any form of cell stress or death, in a way that whole organism deterioration inevitably coincides with loss of respiration. Olatona et al. advise that brain damage can stem from dysfunction of astrocytic glutamate regulation, resulting in hyperexcitation, calcium dysregulation, mitochondrial failure and rupture with resultant astrocytic and microglial neuroinflammation, key events possible in many neurodegenerative diseases and aging. As Liu et al. advise proteinopathies across major neurodegenerative diseases coincide with defective mitophagy and mitochondrial biogenesis, we further highlight the significance of organelle-level aggregation stemming from protein-nucleic acid complexes, and mitochondria-associated endoplasmic reticulum membrane interference, beyond homotypic to protein aggregation from mutation or oxidation induced-misfolding, chaperone dysfunction, or impaired proteasomal/autophagic clearance. The implication of progressive complex cell matter formation and neuroinflammation, on the approach of precision pharmaceutical research is significant. Furthermore, this reinforces our perspective that simply enhancing mitochondrial respiration, supplementation with metabolic precursors or substrates, or antioxidants is unlikely to greatly improve whole organism survival against cumulative genetic or environmental factors in neurodegeneration or aging, even while effectively being secondary mitochondrial diseases (Ng et al., 2024).

The value of reproducing cytopathology prior to significant apoptotic or necrotic events post-mortem *in vitro* remains in absence of real-time human *in vivo* methods. However, whether current cell culture systems with weekly to monthly timeframes can be effective models of insidious late-onset human neurodegenerative factors for therapeutic treatment, remains to be answered. Furthermore, there is a need for studies to clarify valid causative factors and therapeutics, beyond collective ideation with rigorous testing of converse hypotheses, and appreciation of complexity of cumulative organelle-level aggregation, neuroinflammation, genomic and mitochondrial DNA damage. Incidentally, while our topic indicated the prevalence of animal model platforms for conserved or humanized approaches, along with ethical considerations, we look toward human induced pluripotent stem cell culture models of greater relevance in neurodegeneration and aging.
